# Study on the Binding Behavior of Chloride Ion and Ettringite in Nano-Metakaolin Cement by Seawater Mixing and Curing Temperatures

**DOI:** 10.3390/ma17163943

**Published:** 2024-08-08

**Authors:** Zhisheng Fang, Shiyi Zhang, Wenjie Qi, Yingfang Fan, Surendra P. Shah, Junjie Zheng

**Affiliations:** 1 School of Civil Engineering and Geomatics, Shandong University of Technology, Zibo 255000, China; 2 Institute of Road and Bridge Engineering, Dalian Maritime University, Dalian 116026, China; 3 Department of Civil and Environmental Engineering, Northwestern University, Evanston, IL 60208, USA; 4 Department of Civil Engineering, University of Texas at Arlington, Arlington, TX 76010, USA; 5 School of Civil Engineering, Wuhan University, Wuhan 430000, China

**Keywords:** ettringite, chloride ion binding, chlorine content, curing temperature, nano-metakaolin

## Abstract

Mixing cement with seawater will cause the hydration process of cement to be different from that of ordinary cement, which will significantly affect cement’s mechanical properties and durability. This article investigates the effects of chloride ion concentration, curing temperature, and nano-metakaolin content on the evolution process of Friedel’s salts and ettringite (AFt) crystals in cement pastes. The study was conducted using X-ray diffraction (XRD), thermal analysis (TG), scanning electron microscopy (SEM), and mercury-intrusion porosimetry (MIP). The results show that chlorine salt can increase the production of Friedel’s salt and ettringite, and the delayed AFt production increases by up to 27.95% after the addition of chlorine salt, which has an adverse effect on cement-based materials. Increasing the curing temperature and increasing the nano-metakaolin dosage increased the generation of Friedel’s salt and decreased the delayed AFt generation, which resulted in a decrease in the length and diameter of the AFt crystals. After 28 days of high-temperature curing and the addition of nano-metakaolin, Friedel’s salt production increased by 13.40% and 14.34%, respectively, and ettringite production decreased by 9.68% and 7.93%, respectively. Increasing the curing temperature and adding nano-metakaolin can reduce the adverse effect of delayed ettringite increases due to chloride ion binding.

## 1. Introduction

The construction of various infrastructures demands significant concrete and consumes abundant resources such as fresh water, sand, and gravel. As environmental consciousness grows, there is a mounting emphasis on minimizing the use of freshwater and sand resources. To address this concern, efforts have been made to explore using marine resources such as seawater and sand in concrete [1,2], particularly in unreinforced concrete [3,4,5,6]. On the one hand, the use of seawater as mixing water can reduce the demand for freshwater resources, helping to protect local freshwater ecosystems and water supplies. On the other hand, the use of seawater can reduce the energy consumption required for freshwater extraction, treatment, and transportation, which can reduce carbon emissions and other environmental impacts. This is conducive to the sustainable development of the ecological environment.

Seawater and sea sand introduce chloride ions into the cement, influencing cement-based materials’ hydration process and durability. Upon entering the cement, chloride ions dissolve in the pore solution, forming free chloride ions [7]. These free chloride ions can lead to steel-bar corrosion, adversely affecting the concrete structure. Besides free chloride ions, chlorides can combine through physical adsorption and chemical reactions to create bonded chloride ions [8,9]. In the ordinary Portland cement system, physical adsorption largely relies on the highly specific surface area of C-S-H [10]. At the same time, chemical binding involves reactions between chloride ions and tricalcium aluminate (C_3_A) or calcium monosulphoaluminate (AFm) [11], resulting in compounds like Friedel’s salts [7,12]. In cement, AFm contributes the most to the binding of chloride ions, and its mechanism is mainly through the replacement of anions in AFm by chloride ions [13] to form Friedel’s salt. Chloride ion binding can reduce the number of free chloride ions [14,15,16], and Friedel’s salt generated by chloride ion binding changes the pore distribution of cement. Friedel’s salt, as a solid-phase product with large volume, can fill the pores of cement-based materials, reducing porosity. Consequently, the energy from chloride ion binding enhances the longevity of reinforced concrete structures [17,18]. Studies have indicated that in the ordinary Portland cement (OPC) system, chemical binding accounts for 70% of the total chloride ion binding, with physical binding contributing 25–28% [19]. Thus, the chemical bonding of chloride ions is paramount. Within ordinary Portland cement, chloride ion binding is influenced by chloride ion concentration, temperature, and supplementary cementitious materials. The concentration of chloride ions may be one of the most critical factors affecting the binding of chloride ions. Some studies have confirmed that for a particular cement, there is a maximum binding capacity for chloride ions. Below this limit, the higher the chloride ion concentration in the pore solution, the stronger the chloride ion-binding ability [7]. Nano-metakaolin contains a high alumina content, facilitating the formation of significant amounts of Friedel’s salt in a chlorine salt environment. Friedel’s salt plays a crucial role in the chemical binding of chloride ions. Li et al. found [20] that nano-metakaolin can significantly reduce free chloride ions in cement-based materials and improve the durability of cement-based materials.

Chloride ions entering the cement undergo chloride binding and promote cement hydration. These ions can react with calcium ions within the cement to create insoluble solid calcium chloride [21]. This reaction reduces the calcium concentration in the solution, leading to the dissolution of cement particles and an increase in the hydration rate. Studies have indicated that higher chloride ion concentrations in the pore solution can expedite the dissolution of C_3_S and C_3_A in cement [8,22], facilitating the precipitation of calcium and aluminate [9]. Furthermore, chlorides react with aluminates and iron oxides [23] to generate Friedel’s salt within the calcium aluminate phase (AFm phase) family [24]. AFt, an essential hydration product of the aluminate phase, plays a critical role in setting control before cement hardening and influences the early strength development of cementitious materials [25]. Research has demonstrated that during the formation of Friedel’s salt, Cl^−^ replaces SO_4_^2−^ in the AFm structure [13,26]. This displacement leads to the release of sulfate ions into the pore solution, which combines with Ca and Al ions or residual AFm to produce delayed AFt [27]. Delayed AFt in hardened cement concrete can induce paste expansion [28,29], resulting in cracks within the cement paste and aggregate cement-paste interface, significantly reducing strength [30,31,32] and impairing concrete durability [33]. Moreover, studies have highlighted the considerable impact of curing temperatures on AFt formation. High-temperature curing at the onset of cement hydration promotes AFt production. With increasing curing duration, elevated temperatures can prompt the gradual conversion of early-formed AFt into AFm [34]. As a low-cost nanomaterial, the filling effect, nucleation effect, and pozzolanic reaction of nano-metakaolin can promote the hydration of cement and improve the properties of cement-based materials [35]. However, the study of nano-metakaolin on the formation of delayed AFt in the chloride environment is less comprehensive, and further study is needed.

In summary, chloride ion binding can improve the microstructure of cement-based materials, and increasing the curing temperature and adding nano-metakaolin can increase the chloride ion-binding capacity and improve the durability of reinforced concrete. However, chloride ions will bind to the delayed AFt formed in the hardened cement concrete, causing expansion damage. The effects of chloride ion concentration, curing temperature, and nano-metakaolin on the formation of Friedel’s salt and AFt were studied. The study was conducted using methods such as X-ray diffraction (XRD), scanning electron microscopy (SEM), thermogravimetric (TG), and mercury-intrusion porosimetry (MIP). The chemical binding of chloride ions and the formation of AFt can be obtained, and the adverse effects of a delayed AFt can be improved.

## 2. Experimental Section

### 2.1. Raw Materials

**Figure 1:**
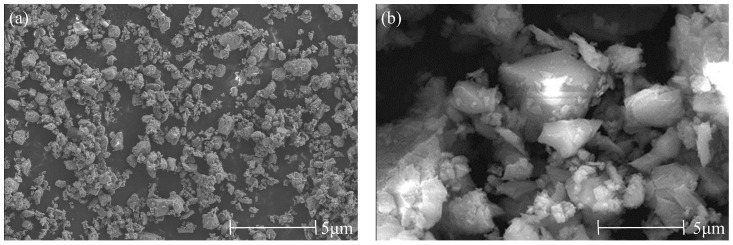
Microstructure of cement and NMK. (**a**) Cement, (**b**) NMK.

The cement used is P·O42.5 ordinary Portland cement produced by Shanshui Dongyue (Zibo, China), and the clay used is nano-metakaolin (NMK) (Wuhan, China). The chemical composition of raw materials determined by X-ray fluorescence analyzer (Rigaku, Tokyo, Japan) is shown in Table 1. The microstructure of raw materials obtained by scanning electron microscopy (Thermo Fisher Scientific, Shanghai, China) is shown in Figure 1.

### 2.2. Specimen-Matching Ratio and Production

#### 2.2.1. Mix Ratio of Specimens

In order to investigate the effects of curing temperature, chloride concentration, and nano-metakaolin content on the chlorine-bonded ettringite, the mix ratio was designed. Three kinds of nano-metakaolin were selected: 0, 3%, and 5% (mass fraction). The water-binder ratio of 0.4 was adopted for all specimens. Pure water and a solution of chlorine salts were used for mixing water. Also, a magnetic stirrer to dissolve sodium chloride into water was used, resulting in a chloride ion concentration of 1% and a chlorine salt solution of 2%. The three solutions are called C0, C1, and C2. The specific test mix ratio is shown in Table 2, where NC represents nano-metakaolin and C is chlorine salt. For example, NC3C1, the nano-metakaolin content, is 3% of the mass of the cementing material, and the chloride ion concentration is 1%.

#### 2.2.2. Specimen Preparation Process

**Figure 2:**
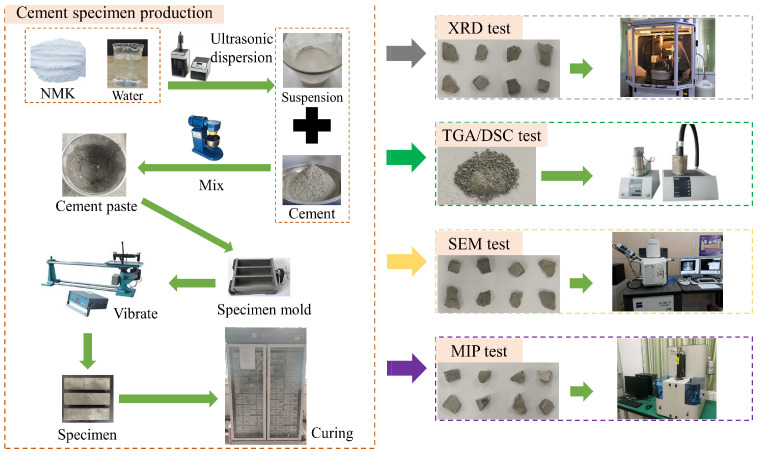
Schematic diagram of specimen production and experimental procedures.

The nano-metakaolin material was dispersed by ultrasonic treatment for 15 min using FS-600N ultrasonic processor (Shengxi ultrasonic instrument, Shanghai, China), and the homogeneous nano-metakaolin material suspension was prepared. The cement paste mixture was prepared by using the cement paste mixer. The prepared cement paste was poured into the mold of 40 mm × 40 mm × 160 mm. Then, the specimen was demoulded after 24 h and cured at 20 °C and 50 °C. Corresponding tests were conducted with curings of 3 days and 28 days, respectively. The specimen preparation process is shown in Figure 2.

### 2.3. Test Methods

#### 2.3.1. XRD

XRD was performed on the 3 days and 28 days aged paste samples. The middle of the cement specimen was sampled and made into a thin slice, which was soaked in anhydrous ethanol for more than 24 h to terminate hydration. The wafer was dried under vacuum at 40 °C for 48 h, and the crystal phase change was analyzed by X-ray diffraction technique. The X-ray diffraction was analyzed by the Panako sharp XRD tester (Malvern panalytical, London, UK). The scanning range was 5°–65°, and the speed was set to 0.02 per step. All samples were subjected to three parallel experiments.

#### 2.3.2. Thermogravimetric Analysis (TG)

TG was performed on the 3 days and 28 days aged paste samples. After grinding the cement samples with mortar, they were sifted through 80 μm fine screen and soaked in anhydrous ethanol for more than 24 h. Then, hydration was terminated and the analytical test samples of cement-based composite materials were prepared under vacuum-drying conditions at 40 °C in an oven. The prepared powder samples were analyzed using a comprehensive thermal analyzer made by TA Instruments. The temperature range was from room temperature of 1000 °C and the heating rate was 10 °C/min. Nitrogen was used as the protection gas for TG analysis. All samples were subjected to three parallel experiments.

#### 2.3.3. Scanning Electron Microscope (SEM)

SEM was performed on the 3 days and 28 days aged paste samples. In this experiment, using TFiS Thermo Scientific Apreo S HiVac field emission scanning electron microscope (Thermo Fisher Scientific, Shanghai, China), the test voltage was 20 kV. In order to reduce the test error, the central part of the broken specimen was selected to make thin slices, soaked in anhydrous ethanol to stop hydration, and the slices were dried under vacuum at 40 °C for 48 h. First, the sample was glued to the sample table with conductive adhesive, and it was sprayed with gold to make it conductive, and then the machine was turned on for observation. All samples were subjected to three parallel experiments.

#### 2.3.4. Mercury-Intrusion Porosimetry (MIP)

MIP was performed on the 3 days and 28 days aged paste samples. The MicroActive-AutoPore V9600 mercury injection instrument (Micromeritics, Shanghai, China) was used to determine the pore structure in cement paste. When preparing the mercury injection sample, the middle of the cement specimen was selected for sampling and the sample was cut into small pieces of ~3 mm–4 mm with scissors. Each sample was used for at least two tests. Immediately after sampling, the hardened cement paste fragments were immersed in anhydrous ethanol to terminate hydration. Before the mercury-injection test, the sample was dried in an oven at 40 °C to constant weight to remove water. All samples were subjected to three parallel experiments.

## 3. Results and Discussion

### 3.1. Effect of Chloride Concentration on Friedel’s Salt and AFt

**Figure 3:**
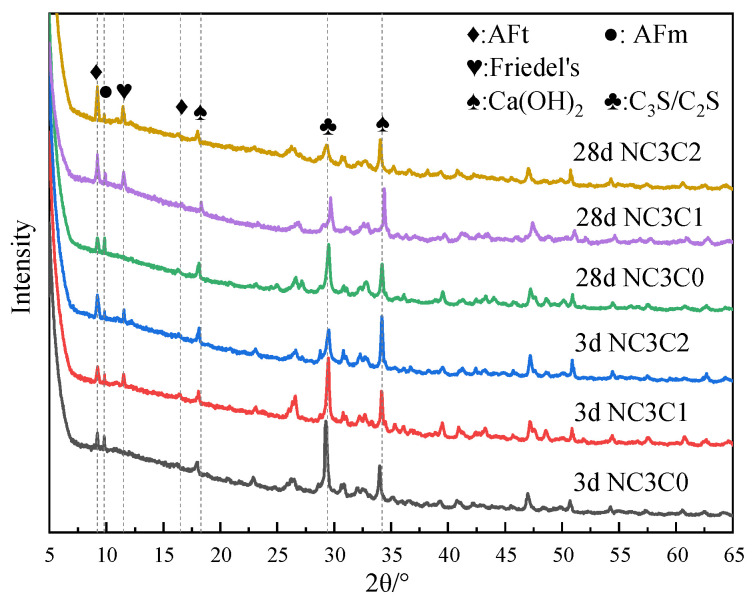
XRD patterns of samples with different chloride content for standard curing.

Figure 3 shows the XRD test results of cement paste with different chloride ion concentrations for curing for 3 days and 28 days with 3% nano-metakaolin. As can be seen from the figure, the main crystal components of hydration products include AFt crystal, AFm, Friedel’s salt, Ca(OH)_2_ crystal, and unhydrated cement clinker minerals (C_2_S and C_3_S). Because C-S-H gel is amorphous, it can not be reflected in the XRD pattern [36,37]. The results show that the diffraction peak of Friedel’s salt appears in the XRD pattern after adding chlorine salt, and the diffraction peak intensity of AFt increases. Within the diffraction peaks of Friedel’s salt, the AFm diffraction peak decreases and the AFt increased with age. The diffraction peak intensity of Friedel’s salt and AFt increased with chloride ion concentration. This shows that chloride ions can increase AFt formation, and the promoting effect is more evident with the increase in chloride ion concentration. This is because chloride ions react with AFm to form Friedel’s salt, which displaces sulfate ions in AFm into the pore solution [27]. When the sulfate ion reaches a specific concentration, it reacts with Ca and Al ions to form AFt. The chemical reaction equation is shown in Equations (1) and (2). According to Equation (1), Friedel’s salt generation will increase with chloride ion concentration, replacing more sulfate ions in the pore solution. This leads to an increase in the concentration of sulfate ions, which triggers a chemical reaction of Formula (2), increasing the amount of AFt (3CaO·Al_2_O_3_·3CaSO_4_·32H_2_O) produced. It is reflected in the XRD pattern where the intensity of the AFt diffraction peak increases. Since AFt is an expansive hydration product, AFt generated at 28 days will cause micro-cracks in the cement paste, adversely affecting the cement.

(1) $$2NaCl+AFm\to Friedel’s+2{Na}^{+}+{SO}_{4}^{2-}+2H_{2}O$$

(2) $$6{\mathrm{Ca}}^{2+}+2\mathrm{Al}{\left(\mathrm{OH}\right)}_{4}+3{\mathrm{SO}}_{4}^{2}+4{\mathrm{OH}}^{-}+26H_{2}O\to 3\mathrm{CaO}\cdot {\mathrm{Al}}_{2}O_{3}\cdot 3{\mathrm{CaSO}}_{4}\cdot 32H_{2}O$$

The weight loss in the temperature range of 50–125 °C in the TG curve is caused by the decomposition of AFt [38,39]. The weight loss of the sample mixed with chloride in the temperature range of 230~410 °C in the TG curve was calculated by subtracting the weight loss of the reference sample [40]. According to Friedel’s salt crystal structure, the primary layer of water consists of six water molecules. Therefore, Friedel’s salt mass fraction (wt.%) can be calculated by the Equation (3) [41]:

(3) $$m_{FS}=\frac{M_{FS}}{6M_{H_{2}O}}m_{H_{2}O}$$

where $m_{FS}$ is the formation mass of Friedel’s salt, $m_{H_{2}O}$ is the mass loss of water in Friedel salt (wt.%), and $M_{FS}$ and $M_{H_{2}O}$ are the molar mass of Friedel’s salt (561.30 g/mol) and water (18.02 g/mol), respectively.

**Figure 4:**
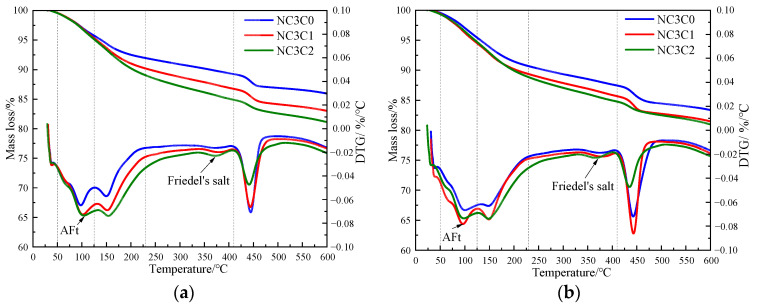
TG curves of specimens with different chloride content for standard curing. (**a**) 3 days, (**b**) 28 days.

**Figure 5:**
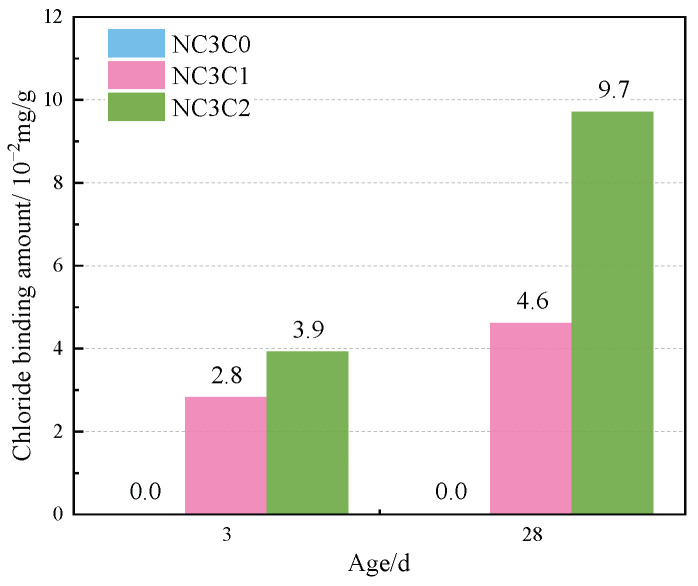
Friedel’s salt production with different chlorine content for standard curing.

Figure 4 shows the TG curves of cement paste with different chloride ion concentrations during curing for 3 days and 28 days when 3% nano-metakaolin is added. The TG\DTG curve shows four peaks. The first peak is near 100 °C, which is the weight loss caused by the dehydration of AFt. The second peak, near 150 °C, is the weight loss caused by the dehydration of C-S-H gel. The third peak is near 360 °C, which is the weight loss caused by Friedel’s salt dehydration. The fourth peak, near 450 °C, is caused by the dehydroxylation of Ca(OH)_2_. The results show that with the increase of chloride ion concentration, the production of Friedel’s salt and AFt gradually increases, and the decomposition peak of AFt crystal in the samples doped with chloride salt slightly shifts to a relatively high temperature. As can be seen from Figure 5, after curing for 3 days, the generated amount of Friedel’s salt in the samples mixed with 1% and 2% chloride salt was 2.8 × 10^−2^ mg/g and 3.9 × 10^−2^ mg/g, and the generated amount of AFt increased by 3.01% and 6.62%, respectively, compared with the samples without chloride ions. With age, the amount of Friedel’s salt and AFt produced in the sample increases. After curing for 28 days, the yield of Friedel’s salt in the specimen mixed with 1% and 2% chloride was 4.6 × 10^−2^ mg/g and 9.7 × 10^−2^ mg/g. After adding 1% and 2% chloride salt, the yield of AFt increased by 26.29% and 27.95%, respectively. This shows that the addition of chloride ions can increase the formation of Friedel’s salt and AFt. It can be found that with the increase of age, the amount of Friedel’s salt generation gradually increases, which will cause more sulfate ions to be released into the pore solution. These sulfate ions react with Ca and Al ions to form delayed ettringite, causing volume expansion, and thus adversely affecting the cement-based materials. This explains the phenomenon of the increase of AFt quantity with the increase of chloride content.

**Figure 6:**
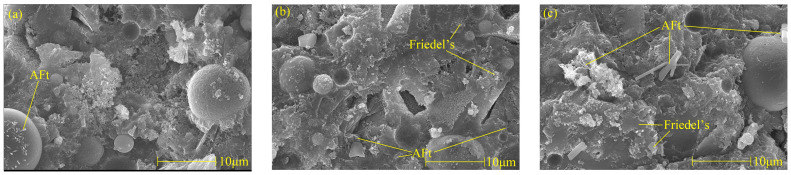
Micromorphologies of Friedel’s salt and AFt with different chlorine content at 28 days. (**a**) NC3C0, (**b**) NC3C1, (**c**) NC3C2.

Figure 6 shows Friedel’s salt and AFt picture of cement paste with different chloride ion concentrations and 3% nano-metakaolin for 28 days. It can be found that with the increase of chloride ion concentration, the hydration products are more closely related, and the cement paste is gradually dense. It can be seen that with the increase of chloride content, the morphology of Friedel’s salts changed significantly, the thickness of plate-shaped Friedel’s salts became thicker, the amount of production increased, and the connection between Friedel’s salts became closer. This phenomenon is consistent with the results of XRD and TG analysis. Therefore, the increase in chloride ion concentration can significantly improve cement-based materials’ chloride ion-binding ability and increase Friedel’s salt production. Through a comparison, it can be found that the number of AFt crystals in the chlorine-doped salt specimens is large, the shape is a typical needle-like crystal, and the aggregation and distribution of AFt are closely related. However, the number of AFt crystals in the samples without chlorine salt is less, the structure of AFt crystals is more regular, and the length of AFt crystals shows a decreasing trend. This is because, on the one hand, chlorine salts promote hydration to increase the formation of AFt, and on the other hand, chloride ions combine to produce delayed AFt. With the increase in chloride content, the number of AFt crystals in the specimen increased, and the diameter and length of AFt crystals showed an increasing trend, with flat edges and sharp angles. The number, length, and diameter of AFt crystals in the specimen with 2% chloride were the largest, while the length and diameter of AFt crystals in the specimen without chloride salt were the most prolonged and minor. After adding chlorine salt, the number, diameter, and length of AFt crystals in the specimen led to the expansion of the cement matrix. This could cause micro-cracks, which harm the hardened cement paste. This is consistent with the results of XRD and TG analyses, and chloride ions increased delayed AFt formation.

The relationship between chloride ion concentration and pore characteristics of hardened cement paste is shown in Table 3. It can be seen that the porosity tends to decrease with the increase of chloride ion concentration. The porosity of cement paste with 1.3% chloride ion concentration decreased by 4.08% and 14.51% for 3 and 28 days compared with the control group. The most probable pore size is the highest proportion of all the apertures of the aperture. The most probable pore size can reflect the distribution of pore size, and the smaller the maximum aperture, the more significant the refinement of pore structure. It can be found that with the increase of chloride ion concentration, the average pore size, the median pore size, and the the most probable pore size show a decreasing trend. This means that with the increase of chloride ion concentration, the cement paste becomes denser and the pore structure is gradually refined. According to the results of XRD, TG, and SEM, delayed AFt production increases gradually with the increase of chloride ion concentration. Because AFt is an expansive hydration product, the denser the pore structure, the greater the internal force generated, which increases the probability of the cracking of the cement base and of decreased durability.

### 3.2. Effect of Curing Temperature on AFt and Friedel’s Salt

**Figure 7:**
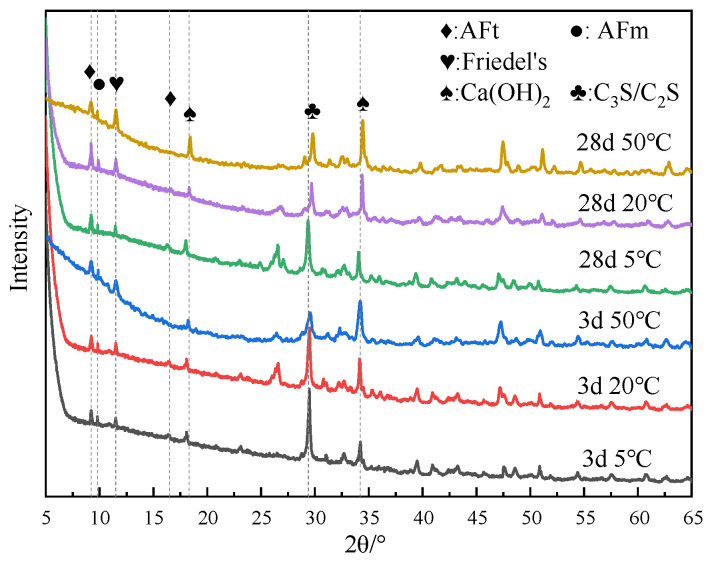
XRD patterns of samples (NC3C1) with different curing temperatures.

Figure 7 shows the XRD test results of specimens doped with 3% nano-metakaolin and 1% chlorine salt at 5 °C, 20 °C, and 50 °C for 3 and 28 days. The results show that the diffraction peak strength of Friedel’s salt increases with the increase of curing temperature and the increase of age. This indicates that the chloride ion-binding ability can be increased with the increase in curing temperature. This is because the increase in curing temperature can promote the hydration of cement and increase the number of hydration products to improve the chloride ion-binding capacity. The diffraction peak intensity of Friedel’s salt decreases at low temperatures. It shows that low-temperature curing can reduce the production of Friedel’s salt. This is due to the slow hydration rate at low temperatures, which reduces the number of hydration products and chloride ion-binding ability. When curing for 3 days, the diffraction peak of AFt gradually increases with the increase of curing temperature. This shows that increasing curing temperature can increase the content of AFt at an early age because the increase of curing temperature promotes the hydration rate of cement and chloride ion bonding and increases the generation amount of AFt. During low-temperature curing, the diffraction peak of AFt gradually decreases, and the decrease in the curing temperature slows down the hydration rate of cement and reduces the generation of AFt. The diffraction peak strength of the AFt crystal of the standard curing specimen was enhanced after 28 days of curing compared with 3 days of curing. This is due to the gradual accumulation of AFt generated by the hydration reaction and the combination of chloride ions, which increases AFt production. The intensity of the AFt diffraction peak of high-temperature curing specimens showed a decreasing trend. The hydration reactivity in cement increased significantly after the temperature increased, and part of the unreacted C_3_A continued to react with the amphibolite formed by cement hydration, decreasing the amount of AFt [42]. To a certain extent, this can avoid the delay of mass formation of AFt in the chloride environment of cement paste and avoid expansion damage. At 28 days, the intensity of AFt diffraction peak of the low-temperature curing specimen increased compared with that of standard curing. This is due to the fact that lower temperatures seem to make AFm unstable (i.e., at low temperatures, AFm content is relatively low). This can provide more aluminum and calcium ions in the pore solution, leading to higher AFt formation at lower temperature exposure.

**Figure 8:**
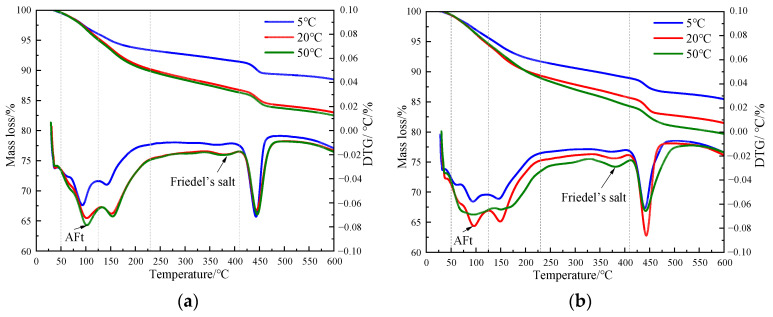
TG curves of specimen (NC3C1) with different curing temperatures. (**a**) 3 days, (**b**) 28 days.

**Figure 9:**
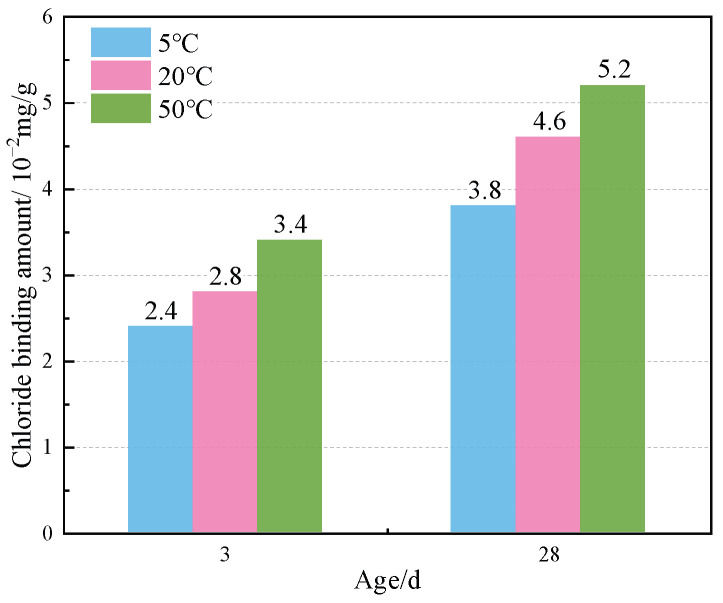
Friedel’s salt production of specimen (NC3C1) with different curing temperatures.

Figure 8 shows the TG curves of specimens doped with 3% nano-metakaolin and 1% chlorine salt when cured at 5 °C, 20 °C, and 50 °C for 3 and 28 days. The phase transitions near 100 °C and 380 °C are the decomposition peaks of AFt and Friedel’s salt, respectively. As can be seen from Figure 9, The results show that after 3 days of curing, the yields of Friedel’s salt and AFt increased by 21.43% and 5.96% compared with that at 20 °C, while the yields of Friedel’s salt and AFt decreased by 14.29%, and the amount of AFt produced increased by 18.20% compared with that at 20 °C. With the increase of age, the yield of Friedel’s salt increased by 13.04%, and the yield of AFt decreased by 9.68%, with the increase in temperature at 28 days. Compared with 20 °C, the yield of Friedel’s salt decreased by 17.39%, and the yield of AFt increased by 8.70%. The results are consistent with those of XRD, which indicate that increasing the curing temperature can reduce the adverse effect of delayed AFt.

**Figure 10:**
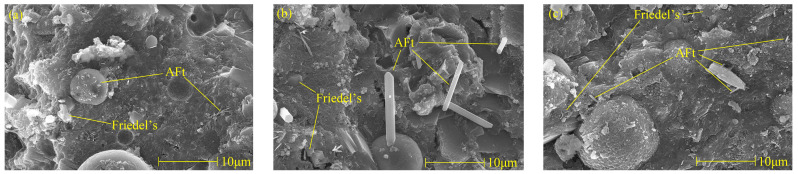
Micromorphologies of Friedel’s salt and AFt with different curing temperature of specimen (NC3C1) at 28 days. (**a**) 5 °C, (**b**) 20 °C, (**c**) 50 °C.

Figure 10 is a picture of Friedel’s salt and AFt when the specimen was cured at 5 °C, 20 °C, and 50 °C for 28 days. It can be found that the cement becomes denser with the increase of the curing temperature. It can be found that there are more pores in low-temperature curing, and the connection between various hydration products is poor. There is a close relationship between the decrease of pore numbers and the increase of hydration products during high-temperature curing. When curing at 50 °C, the plate structure of Friedel’s salt thickened, and the quantity increased. This phenomenon is consistent with the results of XRD and TG analyses. Therefore, it can be concluded that the increase of the curing temperature can increase the production of Friedel’s salt. As can be seen from Figure 10, with the increase of the curing temperature, the hydration reaction continued, and the microstructure of the specimen became denser. By comparing Figure 10b,c, it can be found that the quantity of AFt decreases with the increase of curing temperature on the 28th day of curing, which is consistent with the results of XRD analysis, and the length and diameter of AFt crystals decrease. Therefore, increasing the curing temperature is a way to reduce the harm of delayed AFt.

The relationship between the curing temperature and pore characteristics of hardened cement paste is shown in Table 4. It can be seen that the porosity of the cement paste cured at low temperatures increased, and the porosity of the cement paste cured at 3 days and 28 days increased by 7.73% and 20.37% compared with the control group, respectively. The porosity of the cement paste cured at high temperatures decreased by 30.69% and 22.10% compared with the control group at 3 and 28 days, respectively. This means that with the increase of curing temperature, the pore structure is gradually refined, and the cement paste is denser. With the decrease of the curing temperature, the pore structure is coarsened gradually, and the cement paste becomes looser. According to the results of XRD, TG, and SEM, delayed AFt production increases gradually with the decrease of the curing temperature. At the same time, the pore structure is relatively loose at low temperatures, and the expansion of the internal force generated by AFt has an adverse effect on durability. With the increase of the curing temperature, delayed AFt production decreases, and the length and diameter of AFt crystal decrease. At the same time, the cement paste is dense during high-temperature curing, which promotes the development of the cement’s mechanical properties. Therefore, increasing the curing temperature is a way to reduce the harm of delayed AFt.

### 3.3. Effect of Nano-Metakaolin Content on AFt and Friedel’s Salt

**Figure 11:**
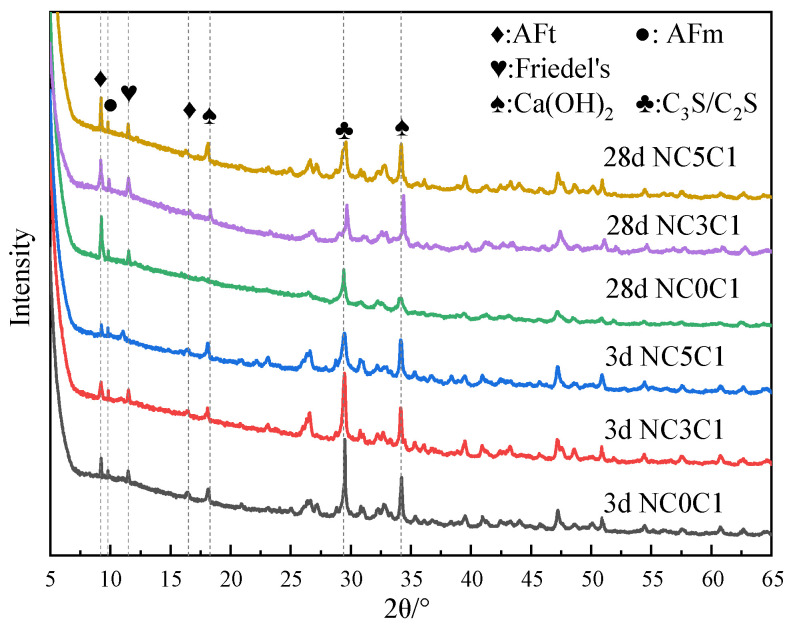
XRD patterns of samples with different nano-metakaolin content for standard curing.

The XRD test results of the specimens with different nano-metakaolin contents for curing for 3 days and 28 days when 1% chloride was added are shown in Figure 11. The results show that the diffraction peak intensity of Friedel’s salt increases, and that of AFt decreases with the increase of nano-metakaolin content at 3 days. This is because the activated alumina in nano-metakaolin reduces the ratio of SO_3_/Al_2_O_3_, decreasing the amount of AFt [34]. It can be found that the AFt diffraction peak of the test specimen with nano-metakaolin has a decreasing trend compared with that without nano-metakaolin after 28 days of curing. This shows that nano-metakaolin has little effect on AFt at 28 days. According to the chemical Equation (4), it can be found that the addition of nano-metakaolin increases the aluminum phase in the cement so that more chloride ions occur in the chemical reaction of Equation (3) instead of Equation (1), which can keep the concentration of sulfate ions in the pore solution low. Thus, the delay of AFt generation is reduced.

(4) $$C_{3}A+{Ca\left(OH\right)}_{2}+2NaCl\to Friedel’s+2{Na}^{+}+2{OH}^{-}$$

**Figure 12:**
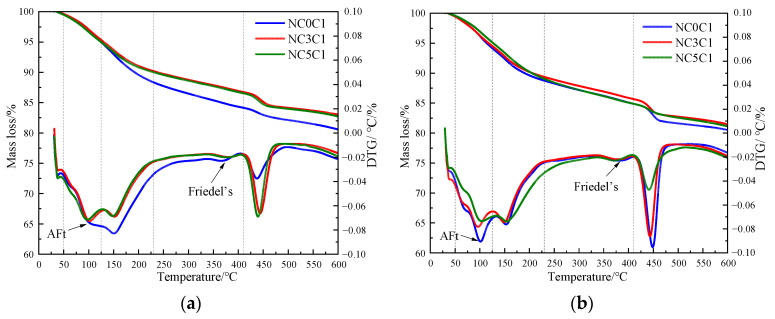
TG curves of specimens with different nano-metakaolin content for standard curing. (**a**) 3 days, (**b**) 28 days.

**Figure 13:**
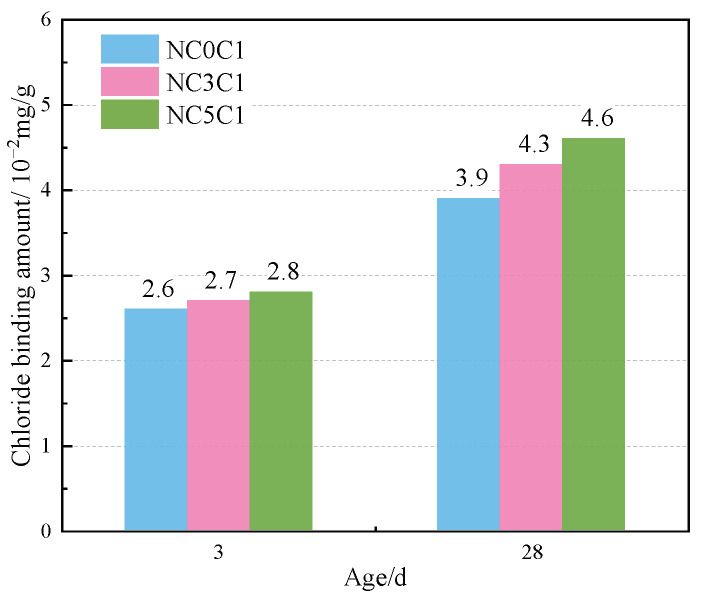
Friedel’s salt production with different nano-metakaolin content for standard curing.

The thermogravimetric (TG) curves of specimens with different nano-metakaolin content and 1% chloride content during curing for 3 days and 28 days are shown in Figure 12. After the early addition of nano-metakaolin, the production of Friedel’s salt increased, but the increment needed to be more prominent. With the increase of age, the production amount of Friedel’s salt increases with the increase of nano-metakaolin content. As can be seen from Figure 13, after curing for 28 days, the yield of Friedel’s salt in the cement mixed with 5% nano-metakaolin was 14.34% higher than without nano-metakaolin. This shows that the aluminum phase rich in nano-metakaolin enhances the chloride ion-binding ability after adding nano-metakaolin. With the increase of nano-metakaolin content, AFt production gradually decreases. This is consistent with XRD results, the addition of nano-metakaolin reduces the generation of delayed AFt. After 3 days of maintenance, the AFt production of the test specimen with 5% nano-metakaolin decreased by 12.69% compared with that without nano-metakaolin. With the increase of age, the AFt production of specimens with 5% nano-metakaolin decreased by 7.93% compared with that without nano-metakaolin at 28 days of curing. This is because the nano-metakaolin is rich in aluminum phase, which can keep the concentration of sulfate ions in the pore solution low, thus reducing the generation of AFt.

**Figure 14:**
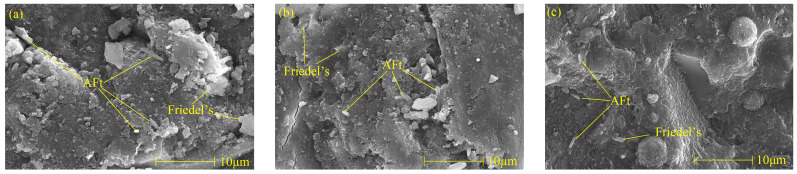
Micromorphologies of Friedel’s salt and AFt with different nano-metakaolin content at 28 days. (**a**) NC0C1, (**b**) NC3C1, (**c**) NC5C1.

The Friedel’s salt and AFt morphology of specimens with different nano-metakaolin contents after curing for 28 days when 1% chlorine was added is shown in Figure 14. With the increase of nano-metakaolin production, cement paste gradually densified. It can be found that with the increase of nano-metakaolin content, the amount of Friedel’s salt increases, and the plate structure becomes thicker, which is closely related to hydration products. This phenomenon is consistent with the results of XRD and TG analysis. Therefore, adding nano-metakaolin can improve chloride ion-binding capacity in cement-based materials. More AFt crystals can be clearly seen in the figure. Through comparison, it can be found that the number of AFt crystals in the samples without nano-metakaolin is large, and the shape is a typical needle-like crystal. However, the number of AFt crystals in the samples doped with nano-metakaolin is small, and the length of AFt crystals shows a decreasing trend. This is because nano-metakaolin keeps the sulfate ions in the pore solution at a lower concentration, thus reducing AFt formation. The number, length, and diameter of the AFt crystals in the specimens doped with 5% nano-metakaolin are the least, and the length and diameter of the AFt crystals in the specimens without nano-metakaolin are the longest and the largest. This is consistent with the results of the XRD and TG analysis. Therefore, adding the appropriate amount of nano-metakaolin can reduce the harm of delayed AFt.

The relationship between nano-metakaolin content and pore characteristics of hardened cement paste is shown in Table 5. It can be seen that the porosity increases after the addition of nano-metakaolin. The porosity of cement paste with 5% nano-metakaolin content increased by 15.77% and 24.84% compared with the control group during 3 and 28 days of maintenance. It can be found that with the increase of nano-metakaolin content, the average pore size, the median pore size, and the most probable pore size show an increasing trend. This means that the increase of nano-metakaolin content will make the cement paste loose and the pore structure gradually coarser. According to the test results of XRD, TG, and SEM, with the increase of nano-metakaolin content, delayed AFt generation gradually decreases, and the length and diameter of AFt crystals decrease. At the same time, the effect of nano-metakaolin on cement pore structure was small at 28 days. Therefore, adding an appropriate amount of nano-metakaolin is a way to reduce the harm of delayed AFt.

## 4. Conclusions

This paper used XRD, TG, SEM, and other experimental characterization methods to study the effects of chloride ion concentration, curing temperature, and nano-metakaolin content on AFt and Friedel’s salt in cement samples. The main findings of this study are summarized as follows:

(1) With the increase of chloride content, Friedel’s salt and AFt production increased. The amount of Friedel’s salt produced in samples with 1% and 2% chloride ion concen-trations was 2.8 × 10^−2^ mg/g and 3.9 × 10^−2^ mg/g at 3 days, and 4.6 × 10^−2^ mg/g and 9.7 × 10^−2^ mg/g at 28 days. At 28 days, delayed AFt production increased by 26.29% and 27.95%, and the length and diameter of ettringite crystals increased. After adding chlorine salt, the porosity of the cement paste was reduced, and the pore size was refined. The denser the pore structure, the greater the internal force caused by delayed AFt, which increases the probability of cement base cracking and adversely affects durability.
(2) Increasing curing temperature can increase the amount of Friedel’s salt and decrease the amount of delayed AFt. After 28 days of high-temperature curing, Friedel’s salt production increased by 13.04%, delayed AFt production decreased by 9.68%, and the length and diameter of the ettringite crystal decreased. Increasing the curing temperature also reduces the porosity of the cement paste and refines the pore size. On the one hand, increasing curing temperature can increase the amount of chlorine-separation bonding. On the other hand, it can reduce the adverse effects of delayed AFt.
(3) The addition of nano-metakaolin can increase the amount of Friedel’s salt and decrease the amount of delayed AFt. When 5% nano-metakaolin was added at 28 days, Friedel’s salt production increased by 14.34%, ettringite production decreased by 7.93%, and the length and diameter of AFt crystals decreased. Adding nano-metakaolin can reduce the formation of delayed AFt and reduce the harm of delayed AFt.
(4) Increasing the curing temperature and adding nano-metakaolin can mitigate the adverse effects of delayed AFt. The effect of seawater mixing on cement hydration reaction is complex. In this paper, only the effect of chloride ions in seawater on the hydration reaction of cement is studied, and the effect of other ions in seawater is ignored. Some factors such as the existence of a certain number of magazines affect the performance of nano-metakaolin. Therefore, more research is needed to understand the effect of seawater on cement hydration and the application of nano-metakaolin in cement-based materials.

## Figures and Tables

**Table 1:** Chemical compositions of the raw materials.

Oxide (wt.%)	CaO	SiO_2_	Al_2_O_3_	Fe_2_O_3_	MgO	Other
Cement	59.31	21.90	6.26	3.79	1.63	7.11
NMK	0.10	46.82	50.46	0.44	0.13	2.05

**Table 2:** Mix ratio of specimens.

Sample	Cement/g	NMK/g	Water/g	Chloride Ion Concentrations/%
NC3C0	485	15	200	0
NC3C1	485	15	200	1
NC3C2	485	15	200	2
NC0C1	500	0	200	1
NC5C1	475	25	200	1

**Table 3:** Pore characteristics of cement paste with different chloride ion concentrations for standard curing.

Sample	Porosity/%	Average Pore Size/nm	Median Pore Size/nm	Most Probable Pore Size/nm
3 days NC3C0	33.07	27.50	59.74	62.51
3 days NC3C1	32.45	22.90	43.58	55.74
3 days NC3C2	31.72	23.43	45.80	55.76
28 days NC3C0	30.88	22.31	41.12	62.48
28 days NC3C1	27.29	20.02	31.81	55.75
28 days NC3C2	26.40	21.24	38.29	69.05

**Table 4:** Pore characteristics of cement paste (NC3C1) with different curing temperatures.

Sample	Porosity/%	Average Pore Size/nm	Median Pore Size/nm	Most Probable Pore Size/nm
3 days 5 °C	34.96	29.64	60.15	55.75
3 days 20 °C	32.45	22.90	43.58	55.74
3 days 50 °C	22.49	13.34	16.22	21.10
28 days 5 °C	32.85	27.08	56.51	55.77
28 days 20 °C	27.29	20.02	31.81	55.75
28 days 50 °C	21.26	10.31	10.00	9.06

**Table 5:** Pore characteristics of cement paste with different nano-metakaolin concentrations for standard curing.

Sample	Porosity/%	Average Pore Size/nm	Median Pore Size/nm	Most Probable Pore Size/nm
3 days NC0C1	28.03	18.05	24.51	40.27
3 days NC3C1	31.96	30.11	62.85	55.77
3 days NC5C1	32.45	22.90	43.58	55.74
28 days NC0C1	21.70	12.55	14.17	5.48
28 days NC3C1	23.07	18.63	29.67	39.02
28 days NC5C1	24.29	20.02	31.81	55.75

## Data Availability

The original contributions presented in the study are included in the article, further inquiries can be directed to the corresponding author.
